# Characterization of continuous bed motion effects on patient breathing and respiratory motion correction in PET/CT imaging

**DOI:** 10.1002/acm2.12785

**Published:** 2019-12-09

**Authors:** Joseph G. Meier, Jeremy J. Erasmus, Gregory W. Gladish, Christine B. Peterson, Radwan H. Diab, Osama R. Mawlawi

**Affiliations:** ^1^ Department of Imaging Physics ‐ Unit 1352 MD Anderson Cancer Center Houston TX USA; ^2^ MD Anderson Cancer Center UTHealth Science Center at Houston Graduate School of Biomedical Sciences Houston TX USA; ^3^ Thoracic Imaging Department ‐ Unit 1478 The University of Texas MD Anderson Cancer Center Houston TX USA; ^4^ Biostatistics Department ‐ Unit 1411 The University of Texas MD Anderson Cancer Center Houston TX USA; ^5^ Faculty of Medicine American University of Beirut Beirut Lebanon

**Keywords:** continuous bed motion, lesion detectability, patient breathing repeatability, PET/CT, respiratory motion correction, SUV quantification

## Abstract

Continuous bed motion (CBM) was recently introduced as an alternative to step‐and‐shoot (SS) mode for PET/CT data acquisition. In CBM, the patient is continuously advanced into the scanner at a preset speed, whereas in SS, the patient is imaged in overlapping bed positions. Previous investigations have shown that patients preferred CBM over SS for PET data acquisition. In this study, we investigated the effect of CBM versus SS on patient breathing and respiratory motion correction. One hundred patients referred for PET/CT were scanned using a Siemens mCT scanner. Patient respiratory waveforms were recorded using an Anzai system and analyzed using four methods: Methods 1 and 2 measured the coefficient of variation (COV) of the respiratory cycle duration (RCD) and amplitude (RCA). Method 3 measured the respiratory frequency signal prominence (RSP) and method 4 measured the width of the HDChest optimal gate (OG) window when using a 35% duty cycle. Waveform analysis was performed over the abdominothoracic region which exhibited the greatest respiratory motion and the results were compared between CBM and SS. Respiratory motion correction was assessed by comparing the ratios of SUVmax, SUVpeak, and CNR of focal FDG uptake, as well as Radiologists’ visual assessment of corresponding image quality of motion corrected and uncorrected images for both acquisition modes. The respiratory waveforms analysis showed that the RCD and RCA COV were 3.7% and 33.3% lower for CBM compared to SS, respectively, while the RSP and OG were 30.5% and 2.0% higher, respectively. Image analysis on the other hand showed that SUVmax, SUVpeak, and CNR were 8.5%, 4.5%, and 3.4% higher for SS compared to CBM, respectively, while the Radiologists’ visual comparison showed similar image quality between acquisition modes. However, none of the results showed statistically significant differences between SS and CBM, suggesting that motion correction is not impacted by acquisition mode.

## 1 INTRODUCTION

One of the unique advances in whole‐body positron emission tomography/computed tomography (PET/CT) is the acquisition of PET data with continuous bed motion (CBM).^1^ Siemens Healthineers (Erlangen, Germany) recently introduced this data acquisition technology on their PET/CT systems with the commercial name of FlowMotion.^2^ Currently, the most common mode of PET data acquisition is step and shoot (SS), which advances the patient into the scanner in incremental steps, with each step being followed by acquisition of PET data while the bed is stationary.

Acquisition of PET data in CBM mode has many advantages over SS mode. For SS acquisition, it is necessary to overlap each bed position to maintain uniform levels of image noise axially. In CBM acquisition, in contrast, the patient passes continuously through the entire PET detector, so all objects are sampled uniformly by the detector. Studies have shown that while there are minimal differences between CBM and SS when measuring the maximum and mean standardized uptake value (SUV) in tumors, the standard deviation of the SUV in both phantom and patient data were higher for SS than for CBM acquisitions.^3^, ^4^ In addition, with CBM, the end‐plane images are acquired in the center of the PET detector, resulting in lower image noise in the most inferior and superior images than that in SS mode which has the lowest sensitivity at these corresponding locations. These improvements in end‐plane image quality have been observed when using CBM in both patient and phantom studies.^2^, ^3^, ^4^ However, an assessment of image quality by radiologists blinded to the acquisition mode demonstrated no consensus in preference for CBM over SS with one study showing a significant preference for CBM images^3^ while the second study showed no significant preference.^4^

Another advantage of CBM is that speed zones can be prescribed in variable lengths to the nearest 0.5 cm, allowing for greater precision, flexibility, and organ‐centric scan prescription than with SS acquisition. In SS mode, a whole extra bed position must be prescribed when the imaged area is slightly larger than the axial extent of the detector. In addition to the resultant increase in acquisition time, any anatomy that does not have to be imaged but is included in the SS PET prescription will be unnecessarily exposed to CT radiation. In one study, researchers compared the CBM scan prescription used in patient examinations with the SS prescription that would have been used for these patients. On average, the scan length was 3.5% shorter and the CT radiation dose was 0.5 mSv lower in CBM mode than in SS mode.^5^ Another important consideration for PET scans is the patient experience, and this was investigated in a randomized crossover study in which patients were scanned in both CBM and SS mode, and the study showed that patients strongly preferred CBM because it has less abrupt motion, is quieter, and is more relaxing.^4^

None of these previous studies, however, evaluated the impact of CBM and SS acquisition modes on patient breathing and the effect these two modes have on respiratory motion correction. Respiratory motion blur in PET/CT can cause a multitude of challenges, including decreased tumor detectability, underestimation of radiotracer concentrations, and misalignments with anatomical images in areas affected by respiratory motion.^6^, ^7^ Although numerous methodologies have been developed to correct for respiratory motion blur,^8^, ^9^, ^10^, ^11^ several studies have demonstrated that the regularity of patient breathing patterns can greatly impact the efficacy of PET images both without and with respiratory motion correction.^12^, ^13^, ^14^

In this study, we prospectively assessed the impact of CBM and SS acquisition modes on the regularity of patient breathing and quantitative and visual assessment of respiratory motion corrected images in PET/CT. To the best of our knowledge, this is the first time such an investigation has been performed. Should CBM or SS produce patient breathing with more regularity and consequently higher quality of motion corrected images, this would provide valuable information on which acquisition mode is superior for respiratory motion corrected studies in PET/CT.

## 2 METHODS

### 2.1 Patients

In total, 100 patients with varying disease stages referred for PET/CT imaging were recruited for this study. 50 patients were assigned to CBM mode, while 50 others were assigned to SS mode. To ensure even distribution between the BMI ranges in both groups, patients were recruited to fill five body mass index (BMI) ranges (BMI < 20, 20 ≤ BMI<25, 25 ≤ BMI<30, 30 ≤ BMI<35, and BMI ≥ 35) at 10 patients per range.

The CBM and SS modes were assigned randomly, and patients were blinded to the acquisition mode. A total of 57 male and 43 female patients were scanned (mean age, 56.2 ± 13.7 years; mean BMI, 28.7 ± 8.7). Contrast CT studies can be very unpleasant for patients, so to reduce potential variables which could influence patient breathing, only patients undergoing non contrast CT scans were recruited. Patients fasted before injection of 352.2 ± 39.8 MBq ^18^F‐fluorodeoxyglucose. The mean ± SD time from injection to the start of PET acquisition was 68.5 ± 8.9 min. This study was approved by the Institutional Review Board (IRB 2015–0986), and all patients gave written informed consent to participate prior to imaging.

### 2.2 PET/CT Acquisition and image reconstruction

All patients were scanned using a four‐ring Biograph mCT Flow system (Siemens Healthineers), which was previously characterized.^15^ A free‐breathing helical CT scan was acquired for attenuation correction and anatomical localization using CARE Dose4D (quality reference 90 mAs), CARE kV (quality reference: 120 kV), a 16 × 1.2 mm detector configuration, and a pitch of 1.4. For the PET acquisition, clinical CBM table speeds and equivalent SS bed times were prescribed based on BMI. For BMIs less than 40, scans from the top of head to the pelvis were acquired at 1 mm/s (2.3 min/bed), and scans of the lower extremities were acquired at 1.5 mm/s (1.5 min/bed). For BMIs greater than or equal to 40, these values were 0.8 mm/s (2.8 min/bed), and 1.5 mm/s (1.5 min/bed) respectively. For both modes of acquisition (CBM and SS), patient respiratory waveforms were recorded throughout the whole‐body scanning using an AZ‐733V respiratory gating system (Anzai Medical, Tokyo, Japan) with the Anzai load cell fixing belt placed between the xyphoid process and umbilicus. All respiratory waveforms were acquired using the Siemens Healthineers PET/CT software interface, which allowed for determination of when the SS table transition times occurred. PET image reconstruction was performed with and without motion correction using: 2 iterations, 21 subsets, time‐of‐flight information, point‐spread function correction, 200 × 200 matrix, 4.07 mm × 4.07 mm × 2.03 mm voxel size, and 5‐mm full width at half‐maximum isotropic Gaussian post‐reconstruction filter. Reconstruction without motion correction for both acquisition modes (CBM and SS) will here onwards be referred to as static whole body (SWB), while reconstructions with motion correction for both acquisition modes (CBM and SS) were performed using a recently introduced respiratory motion correction algorithm (OncoFreeze) that utilizes elastic motion deblurring (EMDB).^9^

### 2.3 Respiratory waveform analysis

To evaluate the impact of the CBM and SS acquisition modes on the regularity of patient breathing, we analyzed the quality of the corresponding patient respiratory waveforms using multiple approaches. All analyses were performed at the location of the fourth bed position, as this location is most likely to cover the lower lung to upper abdominal area where respiratory motion blur is most severe. For waveform analysis corresponding to CBM acquisitions, we selected a segment of the waveform that came from an equivalent time period as bed four for the SS patients, based on the BMI‐dependent acquisition time of the SS protocol (t = 6.9:9.2 min for BMI < 40, t = 8.4:11.2 min for BMI ≥ 40).

**Figure 1:**
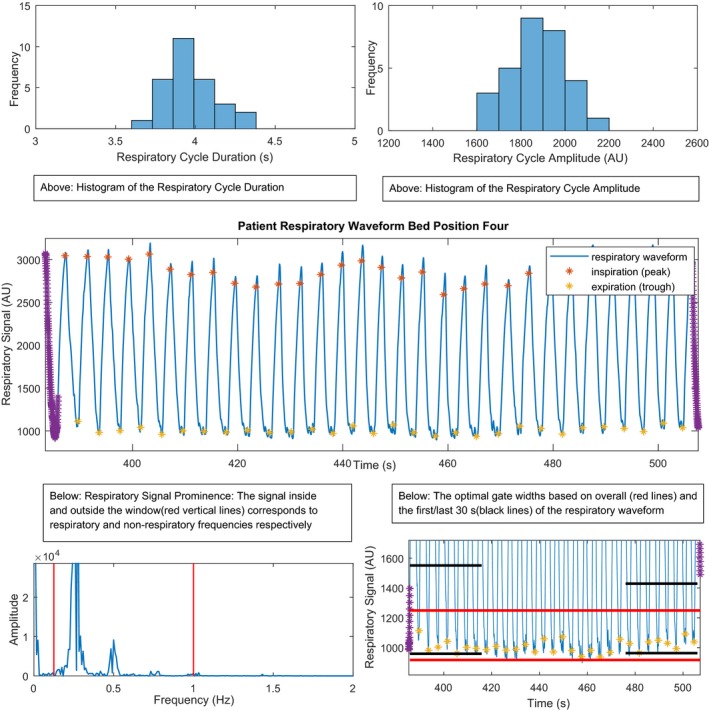
An example patient respiratory waveform at bed position four along with the four different analysis techniques.

The regularity of the patient respiratory waveforms acquired during CBM and SS data acquisition was determined using four analysis techniques. The first and second analysis techniques measured the coefficient of variation (COV) of the respiratory cycle durations (RCD) and the respiratory cycle amplitude (RCA) (Fig. 1) for the CBM and SS patient cohorts and the results were compared between the two acquisition modes. Patients with very repetitive breathing will have the lowest coefficients of variation for these values. The third technique calculated the respiratory frequency range signal prominence (RSP).^16^ The RSP calculates the ratio of the energy spectral density of the signals within a respiratory frequency range corresponding to human breathing to the energy spectral density of the signals outside this range, usually attributed to nonrepetitive respiratory breathing and signal noise (Fig. 1). Based on observations of our patient population, we defined the human respiratory frequency range as 0.1–1.0 Hz (1–10 s). A high RSP is indicative of a waveform that is more repetitive than one with a lower RSP. RSP data for both CBM and SS patient cohorts were then compared. Finally, in the fourth technique, the optimal gate (OG) width was calculated according to the HDChest algorithm with 35% of the breathing signal falling within this amplitude width.^8^ All OG widths were normalized to the same amplitude range. The OG width for patients with nonrepetitive breathing should be wider and cause more respiratory motion blur than that for patients with repetitive breathing who consistently return to the same end‐of‐expiration location in the breathing cycle (Fig. 1). OG width data were then compared between the two acquisition modes (CBM and SS). For all analysis methods, the measurements were summarized by their medians [interquartile range] and the percent changes in the medians between the two data acquisition modes (CBM and SS) were calculated with respect to the SS medians.

To assess the impact of abrupt SS transitions between bed positions immediately following the transitions, the above four waveform analyses were repeated for the first 30 s of bed position 4 and the results were compared with those for the last 30 s of that bed position. The comparisons were performed on waveforms that were acquired within the CBM and SS groups independently. All measurements comparing the first and last 30 s were summarized by their medians [interquartile range] and the percent changes in the medians were calculated with respect to the first 30 s medians.

### 2.4 Respiratory motion quantification and image quality assessment

To assess the impact of CBM and SS on image quantification and quality of motion corrected images, we measured SUVmax, SUVpeak, and CNR on various foci of FDG uptake (tumor, kidney medullae, spleen, and gastrointestinal). Since this is a non‐crossover study where we cannot directly compare quantitative measurements from CBM to those of SS, we calculated the ratio of each of these metrics in motion corrected images (EMDB) to non‐corrected images (SWB) and compared the results between acquisition modes (SS vs. CBM). The CNR was calculated as:

(1) $$\text{CNR}=\frac{\text{SUVmax},\text{focus}-\text{SUVmax},\text{liver}}{\text{SUV}\;\text{SD},\text{liver}}$$

where the SUV standard deviation (SUV SD) was measured in healthy liver tissue using a 3 cm diameter spherical region of interest. Only a single focus was analyzed for each patient.

All SS image measurements were made on the reconstruction from the fourth bed. To compare similar locations in the chest and abdomen for CBM datasets, only the images from the same axial range as the fourth bed position in SS acquisition were analyzed. All measurements were summarized by their medians [interquartile range] and the percent changes in the medians were calculated with respect to the SS medians.

### 2.5 Physician assessment of image quality

**Figure 2:**
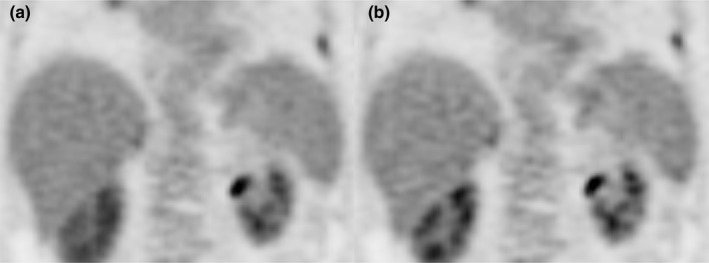
Patient images of the (a) non‐motion corrected SWB and (b) the motion corrected EMDB reconstructions for physician interpretation. The images are from a CBM acquisition. CBM, continuous bed motion; EMDB, elastic motion deblurring; SWB, static whole body.

To assess the impact of CBM and SS acquisition modes on the visual evaluation of image quality, two radiologists experienced in PET/CT interpretation were asked to compare the patients’ EMDB motion corrected images to uncorrected SWB images from bed position four (Fig. 2). In order to compare the two reconstructions, each radiologist was presented with side by side coronal views of the SWB and EMDB reconstructions in a randomized order and only one patient was viewed at a time. The radiologists assessed if there was any difference in motion blur between the two image series. If there was no difference, both image series were assigned a score of zero. If one image series had more blur, then it was scored on a continuous scale (slightly more motion blur (1), moderately more motion blur (2), and significantly more motion blur (3)). Finally, a motion blur score difference was calculated by subtracting the EMDB score from the SWB score for each patient according to equation.2

(2) motion blur score difference=SWBscore-EMDBscore

A positive difference indicates that the SWB images have more motion blur. The acquisition mode (SS or CBM) which results in the highest motion blur score difference indicates that respiratory motion correction was most effective with that mode. The motion blur score differences were summarized by their medians [interquartile range] for the CBM and SS groups respectively. To assess the intrareader reliability of each reader, 20 randomly selected patient studies were repeated.

### 2.6 Statistical analysis

All statistical analyses were performed using the Prism software program (version 7.03; GraphPad Software, San Diego, CA) and the R computing language (version 3.5.0). All waveform characteristics, respiratory motion correction measurements, and respiratory motion blur assessment scores were compared using the Mann–Whitney *U* and Wilcoxon signed‐rank tests for nonpaired and paired data, respectively. To control the false discovery rate due to multiple testing, the Benjamini–Hochberg procedure was used. P values less than 0.05 were considered significant. A two‐way random effects, absolute agreement, multiple raters intraclass correlation coefficient was used to assess interreader reliability regarding the respiratory motion blur scores, whereas a two‐way mixed effects, absolute agreement, multiple raters intraclass correlation coefficient was used to assess intrareader reliability regarding these scores.^17^

## 3 RESULTS

### 3.1 Respiratory waveform analysis

Results of the analysis of patient respiratory waveforms throughout the entire duration of bed position four are shown in Table 1. The results show that CBM has a lower COV for RCD and RCA but larger values for RSP when compared to SS. The results also show that while RCD COV and OG had small percent changes (<5%) in median values between the two acquisition modes (CBM and SS), those for RCA COV and RSP had larger percent changes (>30%). However, none of these results were statistically significant.

**Table 1:** Comparison of the four different analysis methods between CBM and SS for the entire waveform duration for bed position four.

Measurement	Scan Mode	Median [interquartile]	% change in median	*P*‐value
RCD COV	SS	0.28 [0.18]	3.7	0.86
	CBM	0.27 [0.21]		
RCA COV	SS	0.32 [0.32]	33.3	0.55
	CBM	0.24 [0.21]		
RSP	SS	5.57 [9.04]	−30.5	0.86
	CBM	8.02 [7.3]		
OG	SS	429.5 [293]	−2.0	0.55
	CBM	438.4 [273.2]		

Abbreviations: CBM, continuous bed motion; COV, coefficient of variation; OG, optimal gate; RCA, respiratory cycle amplitude; RCD, respiratory cycle duration; RSP, range signal prominence; SS, step‐and‐shoot.

Analysis of patient respiratory waveforms of the first 30 s of bed position four in comparison to the last 30 s of bed position four are shown in Table 2. The table shows that most results of the first 30 s were lower in comparison to the last 30 s for both the CBM and SS acquisition modes, respectively. The results also showed that the corresponding COV values for CBM were mostly lower than SS while the RSP values were higher. However, the OG results showed an opposite effect in that the CBM OG width was higher. Overall, however, none of these results showed statistically significant differences between the first 30 s and the last 30 s of bed position four for both CBM and SS modes of PET acquisition respectively.

**Table 2:** Waveform analysis of the first and last 30 s of bed position four.

Measurement	Time Analyzed	Median [interquartile]	% change in median	*P*‐value
SS RCD COV	BEG_30s	0.11 [0.19]	−47.6	0.17
	END_30s	0.21 [0.24]		
CBM RCD COV	BEG_30s	0.14 [0.22]	−12.5	0.98
	END_30s	0.16 [0.24]		
SS RCA COV	BEG_30s	0.2 [0.3]	−23.1	0.98
	END_30s	0.26 [0.4]		
CBM RCA COV	BEG_30s	0.15 [0.21]	−25.0	0.69
	END_30s	0.2 [0.2]		
SS RSP	BEG_30s	5.6 [7.6]	−13.8	0.15
	END_30s	6.5 [9.3]		
CBM RSP	BEG_30s	6.7 [7.7]	−10.7	0.71
	END_30s	7.5 [7.9]		
SS OG	BEG_30s	362.4 [287.4]	1.5	0.98
	END_30s	357 [254.6]		
CBM OG	BEG_30s	405.8 [299.8]	−1.9	0.98
	END_30s	413.6 [327.9]		

Abbreviations: CBM, continuous bed motion; COV, coefficient of variation; OG, optimal gate; RCA, respiratory cycle amplitude; RCD, respiratory cycle duration; RSP, range signal prominence; SS, step‐and‐shoot.

### 3.2 Respiratory motion quantification and image quality

As seen in Table 3, the ratios of EMDB to SWB measurements for SUVmax, SUVpeak, and CNR for both acquisition modes (CBM and SS) indicated that the EMDB reconstruction improved foci quantification (ratios >1), and detectability (ratios >1). However, while the SS ratios were consistently higher than CBM, none of the differences in these ratios were statistically significant.

**Table 3:** Results for the motion quantification measurements. The scores are summarized as the median [interquartile].

Measurement	Scan Mode	Median ratio [interquartile]	% change in median ratio	*P*‐value
SUVmax	SS	1.28 [0.2]	8.5	0.11
	CBM	1.18 [0.21]		
SUVpeak	SS	1.15 [0.12]	4.5	0.11
	CBM	1.1 [0.1]		
CNR	SS	1.23 [0.41]	3.4	0.63
	CBM	1.19 [0.36]		

Abbreviations: CBM, continuous bed motion; SS, step‐and‐shoot.

### 3.3 Physician assessment of image quality

**Figure 3:**
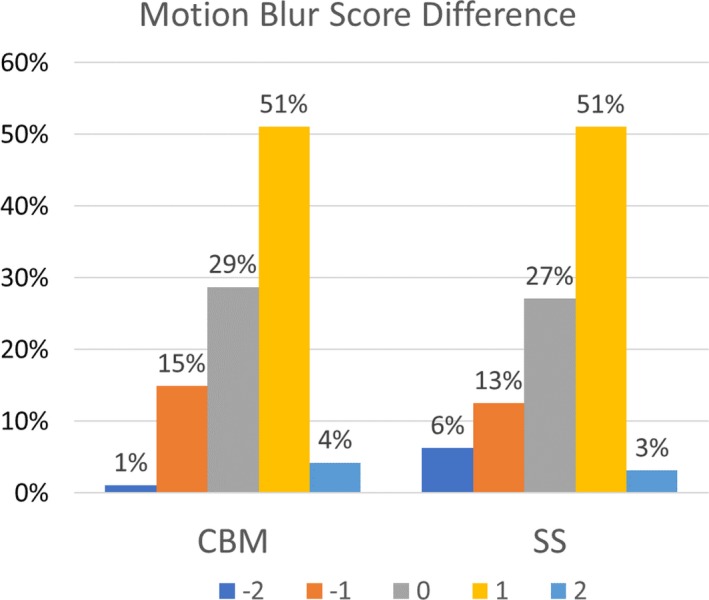
Bar plot of the frequencies of the motion blur score differences for the CBM and SS motion blur visual analysis. CBM, continuous bed motion; SS, step‐and‐shoot.

The physician median [interquartile] overall score differences were 1[1] and 1[1] for CBM and SS mode, respectively (*P* = 0.64). Figure 3 shows the distribution of physician responses of motion blur for SS and CBM. The interreader intraclass correlation coefficient was 0.10 indicating poor reliability (95% confidence interval, −0.34: 0.39). The intrarater intraclass correlation coefficients were −0.34 and 0.24 indicating poor reliability for both readers.

## 4 DISCUSSION

In this work, we investigated whether CBM and SS acquisition modes impact patient breathing and consequently respiratory motion correction during PET/CT. To our knowledge, this is the first comparison study of the impact of these two acquisition modes on patients’ respiratory waveforms and on respiratory motion correction. Our results showed that there is no statistically significant difference in patient breathing when PET data is acquired in CBM vs SS. Furthermore, our results showed that there was no statistically significant difference in quantitative and qualitative evaluation of motion corrected PET images when the data is acquired in CBM vs SS.

We analyzed the patient breathing waveforms using four different methods to capture all potential factors that could affect the repeatability of a patient respiratory waveform when PET data is acquired in CBM and SS. Our first overall patient respiratory waveform assessment was based on measuring the COV of the RCD. If a PET acquisition mode perturbs a patient’s breathing, then the repetitiveness of the cycle duration would change, and this would be shown through an increased COV. This analysis, however, does not differentiate between normal (0.1–1.0 Hz) and abnormal respiratory frequency ranges. For this reason, we used the RSP^16^ which calculates the ratio of signals associated with normal breathing frequencies to those signals associated with abnormal frequencies. High ratios indicate repetitive breathing cycles, while smaller ratios indicate less repetitive breathing. Both of these approaches (RCD and RSP), however, do not capture any information about the breathing cycle amplitudes. For example, a patient breathing waveform might have a consistent RCD but widely varying amplitudes. In this regard, we used RCA as an additional approach to assess the repeatability of the patient’s respiratory cycle. Here also if a PET acquisition mode perturbs the patient’s breathing, then the repetitiveness of the cycle amplitudes will change and this would be shown through an increased COV. Finally, the choice of the OG width as another measure of the breathing cycle repetitiveness was based on its utilization in the HDChest and OncoFreeze (EMDB) motion correction techniques. For patients with less repetitive respiratory waveforms, the OG width will be wider in comparison to more repetitive waveforms. A larger OG width is indicative of poor image quality, as it includes more respiratory motion blur in the motion corrected images.

Although the respiratory waveform analyses (RCD, RSP, RCA, and OG) over the entire duration of bed position four showed no statistical difference between CBM and SS (Table 1), we found that the RCD COV and OG percent changes in the median between SS and CBM were very small, while the percent changes in RCA COV and the RSP were much larger. This shows that although the RCD and OG characterize certain attributes of the waveform, the RCA COV and RSP analyses captured additional information that would not have otherwise been characterized, as shown through the larger percent changes measured for these metrics. The lack of statistically significant differences in all of these cases could be explained by the possibility that any perturbations to the patient breathing that could occur immediately after the SS table transition quickly subside and are averaged out over the entire bed position time frame.

Our analysis comparing the first and last 30 s of bed position four was specifically conducted so that any perturbations that might occur after the table transition will not be averaged out over the entire bed position. Our results, however, showed that also in this case there were no statistical differences in patient breathing between the first and the last 30 s for each of the acquisition modes. After the bed transition for the SS acquisition mode, we expected that all results except for the RSP would be higher in the first 30 s of bed position four due to patient breathing becoming non‐repetitive as a result of the table transition; however, these values were all unexpectedly lower than the last 30 s, except for the SS OG width. One potential explanation is that the SS table transition actually improves the repetitiveness of the patient breathing, a situation that needs further investigation in a future study. For CBM, in contrast, we expected to see negligible differences between the first 30 s and last 30 s due to the smooth motion of the bed throughout the CBM acquisition; however, our results in Table 2 showed that for all of the analysis methods, the first 30 s had lower results than the last 30 s. However, none of these results were statistically significant. Based on all of these results, we conclude that the mode of table motion has no statistically significant impact on the patient breathing.

From the outcomes of the respiratory waveform analysis, it was not expected that the results of the respiratory motion quantification and image quality assessment would be different between the CBM and SS modes. This expectation agrees with our results which showed that although the median SS ratio was higher than the CBM ratio for SUVmax, SUVpeak, and CNR, these differences were not statistically significant. It is important to note, however, that for the F18‐FDG foci that were analyzed, respiratory motion correction for both CBM and SS improved quantification and detectability as shown by the results of the ratio ( >1) of EMDB to SWB for SUVmax, SUVpeak, and CNR respectively in Table 3.

Given the results of the respiratory waveform analysis and the respiratory motion quantification, it was also not expected to observe a statistically significant difference between the respiratory motion blur reduction of CBM and SS as determined by the radiologists. Our results support this expectation. The median motion blur score difference was 1 and 1 respectively for CBM and SS, showing that the EMDB algorithm has a slightly perceivable reduction in motion blur in comparison to SWB; however, there was no statistical difference between the two acquisition modes. Both the inter‐ and intrareader ICC scores were poor for the physician assessment. One potential reason for this result is the slight perceivable difference in respiratory motion blur between images acquired in CBM and SS.

One limitation of this study is that most of the patients (89 of 100) in this study were scanned on an average of four times, and the majority had their last PET/CT scan at MD Anderson. These patients were aware of what to expect during a PET/CT examination, so they had much less anxiety due to fear of the unknown of being in a PET/CT scanner which could affect the regularity of their breathing independent of the acquisition mode than patients undergoing PET/CT for the first time.

Another potential limitation of this study is that it was designed as a non‐crossover study. Such a study design requires a large number of patients to achieve statistical significance. Our study might not have been statistically powered with the number of patients scanned (50 patients per group) to detect differences between the two acquisition modes (CBM vs SS) for the respiratory waveform analysis, respiratory motion correction image quantification and visual assessment of motion blur score. Crossover studies require fewer patients to detect changes between two methods. However, to perform a crossover study design would require scanning the patients in both CBM and SS, thereby doubling the acquisition time, which would have been very challenging to achieve in our very busy clinic. In addition, a challenge with performing this work in a crossover study is that the patient would have to lie on the bed for twice the amount of time, which would likely influence their breathing and consequentially the motion corrected images.

An additional limitation of the study is that we did not recruit patients specifically with lung or liver tumors in areas impacted by respiratory motion. For this reason, several patients had no lesions impacted by respiratory motion, and this is why we analyzed foci in other organs which unavoidably have varying degrees of motion perhaps less than tumors in the lung or liver. In addition, it is easier to visually assess if there is motion blur in smaller and isolated nodules, rather than assessing small structures of an organ such as the heart or the kidney, and this might have limited the radiologists’ perception of respiratory motion blur if it was present in the images.

## 5 CONCLUSION

This study is the first to investigate and consequentially find that the choice of CBM or SS acquisition mode has no statistically significant impact on patient breathing, lesion quantification and detectability, or perceived respiratory motion blur during PET/CT examinations, suggesting that motion correction is not impacted by acquisition mode.

## CONFLICT OF INTEREST

This work was supported in part by a research grant from SIEMENS Healthineers.
